# Supplementary material for: Content-rich biological network constructed by mining PubMed abstracts
Source: BMC Bioinformatics. 2004 Oct 8;5:147. doi: 10.1186/1471-2105-5-147 (PMC528731; doi:10.1186/1471-2105-5-147)
Supplement: Additional File 2 — The original results of the above study (non-essential files are deleted to keep the file size under the limit set by BMC bioinformatics). [file 1471-2105-5-147-S2.bz2 › chilibotAdditionalFile2/dip50/561ID11278498E10922/html/INS_INSR.html]

 


 **INS** and **INSR** 
  
Found 6349 abstracts in PubMed, retrieved 50.  
 

 What does Google say? 
 PDF only 
| .edu only 

---

**Interactive relationship** (e.g. stimulation, inhibition, etc)

**Stimulatory relationship**- In addition to mutant alleles conferring an increased risk of diabetes, these studies have uncovered mutations that protect against insulin  [ **INS** ]  resistance caused by the  **Insr**  mutation, thus providing proof of principle for the notion that certain alleles may confer resistance to diabetes.  Ref: 12788928 J Biol Chem< MedlineTA>J Biol Chem,
**Neutral relationship**- Studies of mice with tissue specific ablation of  **Insr**  function have indicated that both canonical e.g., muscle and adipose tissue and non canonical e.g., liver, pancreatic b cells and brain insulin  [ **INS** ]  target tissues can contribute to insulin  [ **INS** ]  resistance, albeit in a pathogenetically distinct fashion.  Ref: 12788928 J Biol Chem< MedlineTA>J Biol Chem,
- Furthermore, experimental crosses of  **Insr**  mutants with mice carrying mutations that affect insulin  [ **INS** ]  action at more distal steps of the insulin  [ **INS** ]  signalling cascade have begun to unravel the complex genetics of type 2 diabetes.  Ref: 12788928 J Biol Chem< MedlineTA>J Biol Chem,

**Non-interactive relationship** (e.g. studied together, co-existance, homology, etc.)

- Mice bearing targeted gene mutations that affect insulin  [ **INS** ]  receptor  **Insr**  function have contributed important new information on the pathogenesis of type 2 diabetes.  Ref: 12788928 J Biol Chem< MedlineTA>J Biol Chem,
